# Supplementary material for: Obesity Affects Maternal and Neonatal HDL Metabolism and Function
Source: Antioxidants (Basel). 2023 Jan 14;12(1):199. doi: 10.3390/antiox12010199 (PMC9854613; doi:10.3390/antiox12010199)
Supplement: Supplementary file 1 [file antioxidants-12-00199-s001.zip › antioxidants-2132655-Supplementary Material.pdf]

antioxidants

MDPI

Obesity Affects Maternal and Neonatal HDL Metabolism and Function

Supplementary Material

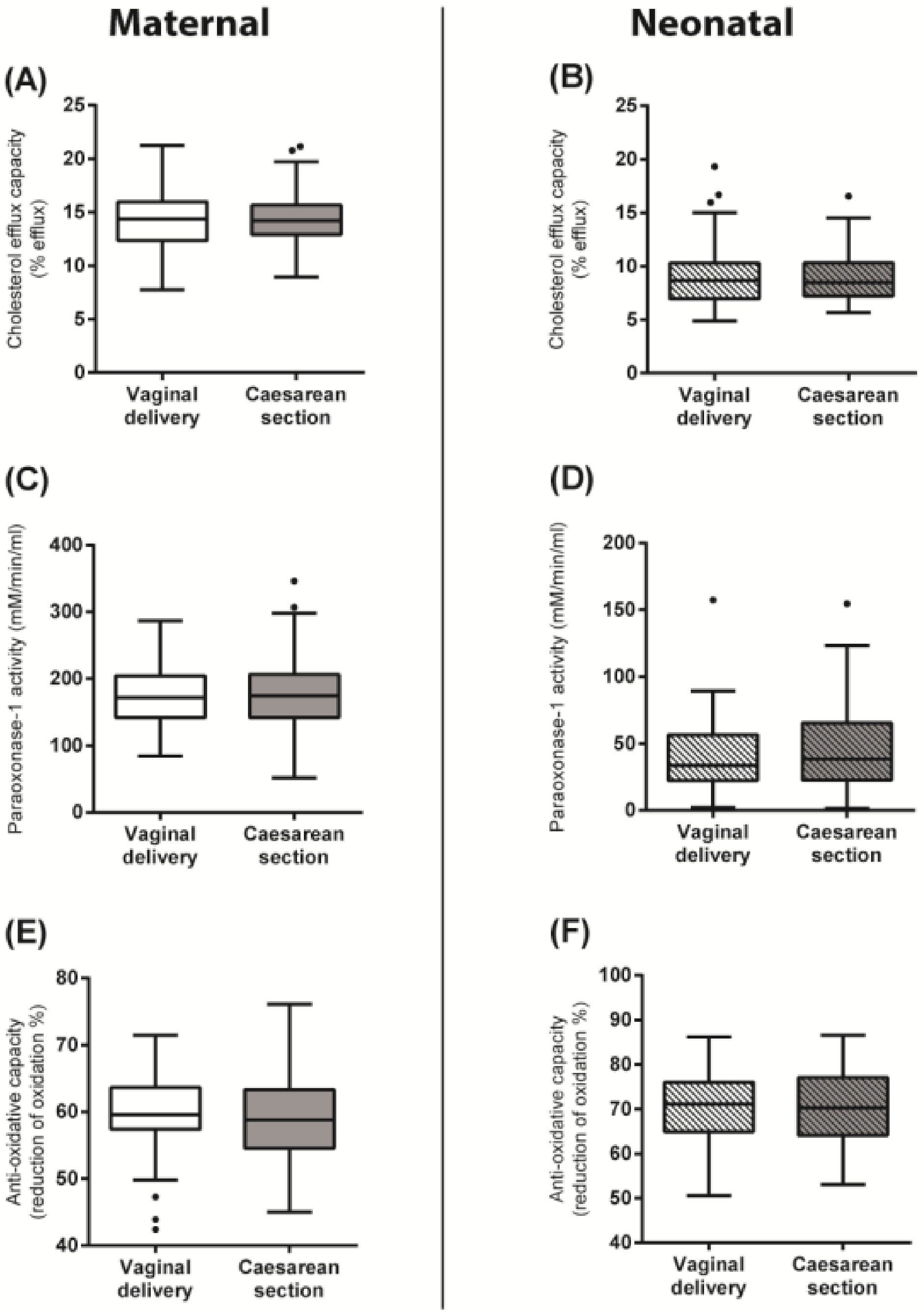

Figure S1. Effect of delivery mode on parameters of HDL function and serum anti-oxidative capacity in mothers and neonates. Cholesterol efflux capacity was assessed with a cell-based assay (A,B). The activity of HDL-associated paraoxonase-1 (PON1) in mothers (C) and offspring (D) was evaluated. Serum antioxidative capacity was assessed in mothers (E) as well as in paired umbilical cord blood (F). Data are presented as Tukey boxplots showing the median and interquartile ranges as well as minimum and maximum values and outliers. Differences were analyzed by ANOVA or Kruskal–Wallis test based on the distribution of the variable.
